# Level and factors associated with preference of institutional delivery among pregnant woman in Debre-tabor town, North West Ethiopia, 2017: a community based cross sectional study

**DOI:** 10.1186/s13104-019-4082-x

**Published:** 2019-01-21

**Authors:** Berhan Tsegaye, Muluesh Abuhay, Edenshaw Admasu, Berhanu Wubale, Kiber Temesgen, Zemenu Yohannes

**Affiliations:** 1 Department of Midwifery, College of Medicine and Health Sciences, Hawassa University, PO Box- 1560, Hawassa, Ethiopia; 2 Department of Midwifery, College of Medicine and Health Sciences, University of Gondar, PO Box-196, Gondar, Ethiopia

**Keywords:** Preference of institutional delivery, Pregnant women, Debretabor town

## Abstract

**Objective:**

Maternal mortality rates in Ethiopia remain highest in the world. Information with respect to factors that influence preference of institutional delivery among pregnant women are of relevance for designing intervention programs to reduce these deaths. This study aimed to determine level and factors affecting preference of institutional delivery among pregnant woman in Debretabor, North West Ethiopia, 2017.

**Result:**

Out of 399 respondents 394 were included in the analysis and making a response rate 98.7%. From a total of 279 (70.8%) respondents prefer health institution as their place of birth. Educational level of unable to read and write (AOR = 0.18, 95% CI 0.06–0.51), Primaryeducation (AOR = 0.25, 95% CI 0.09–0.68), monthly income category of 600–1000 ETB (AOR = 0.24, 95% CI 0.11–0.50), Gravida of more than five (AOR = 0.23, 95% CI 0.08–0.61) and lack of ANC follow up (AOR = 8.33, 95% CI 4–16.6) were significantly affect preference of health institution as place of delivery. Therefore, it is better to give more attention and emphasis on continues education about benefit of institutional delivery, strengthening ANC services and work to improve economic status of women.

## Introduction

Despite in reduction of maternal mortality by 47% in the world by the use of maternal health service, millions of women neglect to use it. Every year, more than 287,000 women die and 12 million suffer from birth complications, but nearly half of all pregnant women in developing countries do not use skilled birth attendants [1]. The maternal mortality ratio in developing countries is 240 per 100,000 live births versus 16 per 100,000 live births in developed countries. Most maternal deaths are avoidable through the use of skilled delivery care. But only 46% of women in low-income countries use it [2]. Globally, about 80% of these preventable maternal deaths are due to severe bleeding, infections, and unsafe induced abortion, hypertensive disorders in pregnancy and obstructed labor [3]. Maternal mortality and morbidity endanger the life of newborn, family and the whole society. It influence health, economic and social aspect of life [4].

Institutional delivery service utilization is a key strategy to reduce maternal death [5]. Ethiopia has achieved still low (26%) proportion of live birth in health facility. Amhara is the fourth least region in the number of deliveries attended in health facilities which accounts only 27.1% [6]. Despite, the expansion of health infrastructure and the introduction of the health Extension program, there are still many barriers preventing women from accessing skill birth attendant (SBA) [7]. Since, every pregnancy faces risk, it is better to assure safe and successful delivery outcomes. It is important to ensure skilled delivery attendant at every child birth [5].

To improve health seeking behavior, it is important to understand factors that influence care-seeking behavior in a given context [8]. A study in jimma town, southwest Ethiopia showed that 35.4% of urban pregnant women still prefer home as their birth place [9]. This shows as a significant number of women in urban settings continue to deliver at home. Women may deliver at health institutions without their preference by shifting their plan of delivery due to complications appear during labor and delivery [10]. A study conducted in South Tigray Zone, among urban resident pregnant women Ethiopia revealed that among 465 pregnant women who planned for institutional delivery, 134 (28.8%) opted out and delivered at their home (missed opportunity) [11].

But most of the previous studies are conducted on institutional delivery utilizations not on their preference. Therefore, this study is used to fill this gap. Neglecting this topic on this subpopulation will cost a lot by missing target points of intervention.

## Main text

### Methods and materials

#### Study settings

A study was conducted in Debretabor town. It is the capital city of South Gondar zone. It is 98 km to the east of Bahirdar the capital of the state. It is about 667 km north of the capital city Addis Ababa. The total population of the town was estimated to be 82,355 of which 40,103 are female population. The town was sub divided by 4 Kebeles. There are a total of 1450 registered pregnant women in Debretabor town, in 2017 [12].

#### Study design and sampling method

A community based quantitative cross sectional study was conducted from July 1 to 30, 2017. The sample size for the study conducted among pregnant women was calculated as 394 using single proportion formula. The inputs of the computation were 95% confidence level, 5% margin of error, expected proportion of preference of delivery place (38.2%) among women’s of child bearing age in Degadamot woreda, northwest Ethiopia, in 2014 [13]. Designed for ordinal logistic regression analysis, the available sample size is judged to be optimal for identifying proportion and factors associated with preference of delivery place with 95% confidence level and 80% power. Simple random sampling was applied to select 394 samples from town health extension worker registration book by lottery method.

#### Population

The source population for this study are all pregnant women in Debretabor town. While the Study population is pregnant women living in Debretabor town and present during study period.

#### Data collection

Data were collected by using pretested, semi-structured, and interviewer administered Questionnaire. The questionnaire was prepared after reviewing relevant literatures. The questionnaire was first developed in English and transferred to local language (Amharic) then back to English to keep its consistency. The Data was collected by 5 BSC female midwives and 5 BSC female nurses after 1 day Training about informed consent, techniques of interviewing, data collection procedures, and different sections of the questionnaire. Two health officers were assigned as supervisors for the data collectors. Overall supervision also made by the principal Investigators.

#### Data analysis

Data were entered and cleaned using Epi Info 7 software and exported to SPSS version 21 for analysis. Bi-variable and multivariable logistic regression were employed. Independent variables less than or equals to 0.2 p-values in the bi-variable logistic regression model were fitted into the multivariable logistic regression model. Variable having *P* value ≤ 0.05 in the multivariable logistic regression analysis was considered as determinant factors of preference of health institutions. The adjusted odd ratios with the 95% confidence intervals were reported. The necessary assumption of logistic regression model was checked by using Hosmer–Lemeshow test of goodness of fit. Descriptive statistics like frequencies and cross tabulation were performed.

### Result

#### Socio-demographic characteristics

From a total of 399 pregnant women recruited 394 participated fully in this study making a response rate of 98.7%. Five (1.3%) of the respondent gave incomplete data and it was discarded. The mean (± SD) age of the respondents were 28.1 ± 5.11 years. Majorities 167 (42.4%) of the pregnant women were within the age range of 25–29 years. Most of the study participants 382 (97%) were followers of orthodox Christianity (Table 1).

**Table 1:** Socio-demographic characteristics of pregnant women in Debretabor town, south Gondar zone, northwest Ethiopia, 2017 (n = 394)

Variables	Categories	N	Percent (%)
Age	15–19	6	1.5
	20–24	87	22.1
	25–29	167	42.4
	30–35	93	23.6
	≥ 35	41	10.4
Religion	Orthodox	382	97.0
	Muslim	12	3.0
Marital status	Married	326	82.7
	Single cohabitated	31	7.9
	Single	9	2.3
	Divorced	16	4.1
	Separated	10	2.5
	Widowed	2	0.5
Ethnicity	Amhara	388	98.5
	Tigre	4	1
	Oromo	2	0.5
Educational status	Illiterate	67	17
	Read and write	41	10.4
	Primary education	125	31.7
	Secondary education	95	24.1
	Tertiary	66	16.8
Husband educational status	Illiterate	31	7.9
	Read and write	45	11.4
	Primary education	74	18.8
	Secondary education	111	28.2
	Tertiary	133	33.8
Women’s occupation	Merchant	48	12.2
	Government worker	69	17.5
	Housewife	234	59.4
	Farmer	8	2.0
	Daily worker	13	3.3
	Student	22	5.6
Husband occupation	Farmer	84	21.3
	Daily worker	64	16.2
	Government worker	108	24.9
	Merchant	98	27.4
	Student	40	10.2
Monthly income	< 320 ETB	20	5.1
	320–600 ETB	9	2.3
	600–1000 ETB	44	11.2
	> 1000 ETB	321	81.5
Total family size	1–5	389	98.7
	> 6	5	1.3

#### Reproductive health factors

Majority 335 (85%) of current pregnancies are planned. Most of the respondents 330 (83.8%) started ANC follow up in current pregnancy. Concerning about time of start of ANC follow up 173 (52.4%) of the respondents start at 3–6 months. Most respondent’s 309 (78.4%) didn’t face any problem in the current pregnancy (Table 2).

**Table 2:** Reproductive factors of preference of pregnant women in Debretabor town northwest Ethiopia, 2017

Variables	Categories	Frequency	Percent
What is the status of current pregnancy?	Unplanned	59	15
	Planned	335	85
Did you begin ANC at current pregnancy?	Yes	330	83.8
	No	64	16.2
How many times did you attend?	1	95	28.7
	2–4	208	63
	> 4	27	8.1
When did you start your ANC attendance?	1–3 months	127	38.4
	3–6 months	173	52.4
	≥ 6 months	30	9
Do you face any problem in current pregnancy?	Yes	85	21.6
	No	314	78.6

#### Health service factors

From a total of 394 respondents 378 (95.9%) of respondents went to health institutions at least one time for ANC or delivery service. Majorities 45% of respondents indicated that bad behavior of professional as the problem during their visit. While other 25.2% face long waiting time to get the service.

#### Magnitude of pregnant women’s preference of delivery place

Out of total of 394 respondents, 279 (70.8%) respondents prefer health institution as their delivery site while the rest 115 (29.2%) prefer home delivery.

#### Factors affecting preference of institutional delivery among pregnant women

Educational status of women was found to be significant factor influencing institutional delivery. Unable to read and write were 18% (AOR = 0.18, 95% CI 0.06–0.51), primary educated were 75% (AOR = 0.25, 95% CI 0.09–0.68) less likely to prefer health institutions as delivery place than tertiary. Average monthly income was another significant factor in this study. Those pregnant women whose average monthly income of 600–1000 ETB were 76% (AOR = 0.24, 95% CI 0.11–0.50) less likely prefer health institution as birth place compared to those of income category of > 1000 ETB. Regarding about the number of pregnancy those respondents whose Gravida greater than 5 were 77% (AOR = 0.23, 95% CI 0.08–0.61) less likely to prefer health institution as delivery site than those of Gravida.1. Concerning about ANC follow up in current pregnancy those who had no ANC follow up were 88% (AOR = 12, 95% CI 0.6–0.25) less likely to prefer health institution as delivery place than those of respondents who had ANC follow up (Table 3).

**Table 3:** Determinants of preference of institutional delivery from backward stepwise (Wald) logistic regression in Debre tabor, North West Ethiopia, July 2017

Variable	HI	Home	COR (95% CI)	AOR (95% CI)	p-value
Educational status					
Unable to read and write	35	32	0.109*	0.183 (0.06–0.51)**	0.001
Able to read and write	25	16	0.156*	0.326 (0.10–1.00)	
Primary education	75	50	0.150*	0.258 (0.09–0.68)**	0.007
Secondary education	84	11	0.76	1.04 (0.34–3.22)	
Tertiary	60	6	1		
Monthly income					
< 320	8	12	0.20*	0.37 (0.11–1.23)	
320–600	4	5	0.24*	0.55 (0.12–2.46)	
600–1000	22	22	0.31*	0.24 (0.11–0.50)**	0.000
> 1000	245	76	1		
Gravidity					
1	186	57	1		
2–5	84	41	0.62	1.11 (0.59–2.09)	
> 5	9	17	0.16*	0.23 (0.08–0.61)**	0.003
Did you attend ANC in current pregnancy?					
Yes	259	71	8*	8 (4–16.6)**	0.000
No	20	44	1		

* Significant for binary variable of p < 0.2

** Adjusted for all significant variables p < 0.05

1: Reference category

### Discussion

This community based study has identified level and factors affecting preference of delivery place among pregnant women in Debretabor town, northwest Ethiopia, in 2017. It revealed that preference of institutional delivery among pregnant women in Debretabor town was 70.8%.

This finding was more than what has been observed in many other country [14, 15]. The possible explanation for this difference may be the difference in study period and study population Moreover, socio-economic characteristic variation which influences positively selection of place of delivery, current strategies of governments for better access to information, awareness about the advantages of institutional delivery by town health extension worker create good media for high proportion of women prefer health institutions as delivery place.

In other ways, this result is in line with the study conducted in South Tigray Zone, Ethiopia. In this study 71.2% of pregnant women preferred to deliver at health institutions [16]. The possible justification for this finding might be in both studies were conducted on urban and pregnant women. In addition, both studies were conducted within 5 years. Regarding about factors associated with preference of delivery place among pregnant women in Debretabor city, some factors were found to influence preference of institutional delivery were lower educational status, low average monthly income, absence of ANC attendance and higher gravidity.

Unable to read and write were 82% and primary educated were 75% less likely to deliver at health institutions than tertiary and above educated. This finding is consistent with previous studies conducted at different area [17, 18]. These might be due the fact that educated women had better awareness about the benefits of institutional delivery. In contrary uneducated women are not familiar with public institutions and feel uneasy about preferring them for delivery. Educated women may accept and utilize new health related information easily. So, they have more decision-making power.

Regarding about average monthly income, those pregnant women whose average monthly income of 600–1000 ETB were 76% less likely to prefer to health institution as birth place than those of 1000 ETB and above. This finding is consistent to the study conducted in Addis Ababa [19]. The possible explanation for this finding may be in principle, health care in Ethiopia for pregnant women is free. However, healthcare facilities seldom run out of stock, requiring a pregnant woman to incur some expenses for supplies or services. Besides, some women must pay transportation fee to travel from home to the health facility. Therefore, pregnant women with good sources of income are better able to pay for transport and unexpected healthcare facility costs. But this finding is inconsistent with findings of the study conducted Benji-Maji zone, southwest Ethiopia in which middle and higher quartile women were 50% and 20% less likely to prefer health institution as birth place as compared than those of lower quartile women [20]. The possible justification for this difference may be most of the women in Benji-Maji were rural and far from health information and less educated than in this study. In general, the effect of economic status on preference of facility-based delivery suggests that the mere provision of services free of charge at point of use is not sufficient to overcome the barriers to access imposed by poverty.

Regarding about ANC attendance those who had ANC follow up were 8 times more likely to prefer health institution delivery than their counterparts. This finding is comparable to previous study [21]. The possible explanation could be women can get information about benefit of institutional delivery and more aware of danger sign of pregnancy and child birth. This could positively shift the preference of pregnant women towards health institution as delivery place.

Regarding about Gravidity those who had 5 and above pregnancy were 77% less likely to prefer institutional delivery than those of primi-Gravida.

### Conclusion

Level of preference of health institution as delivery place was found to be high. Respondent’s educational status, average monthly income, ANC follow up in current pregnancy and gravidity were important predictor of preference of institutional delivery. All stakeholders and policy make should work to alleviate the problem by increasing educational opportunity for all women, expand family planning program in the health institutions and community and Scale up and strengthen ANC follow service for all pregnant women. In addition, improving economic status of women and to boost women’s decision power over income to enhance institutional delivery service utilization.

## Limitation of the study

This study represent only urban pregnant women preference of delivery place. Since, most of pregnant women in Ethiopia reside in rural area. It is better to include urban and rural women in future studies.

## Abbreviations

AOR: adjusted odds ratio
CI: confidence interval
OR: odds ratio
ETB: Ethiopian birr

## References

[1] Dickson KE, Kinney MV, Moxon SG, Ashton J, Zaka N, Simen-Kapeu A, Sharma G, Kerber KJ, Daelmans B, Gülmezoglu AM (2015). Scaling up quality care for mothers and newborns around the time of birth: an overview of methods and analyses of intervention-specific bottlenecks and solutions. BMC Pregnancy Childbirth.

[2] Organization WH. Maternal mortality: to improve maternal health, barriers that limit access to quality maternal health services must be identified and addressed at all levels of the health system: fact sheet. 2014.

[3] Dao B (2012). Guidelines for in-service training in basic and comprehensive emergency obstetric and newborn care.

[4] Mason E, McDougall L, Lawn JE, Gupta A, Claeson M, Pillay Y, Presern C, Lukong MB, Mann G, Wijnroks M (2014). From evidence to action to deliver a healthy start for the next generation. Lancet.

[5] Memirie ST, Verguet S, Norheim OF, Levin C, Johansson KA (2016). Inequalities in utilization of maternal and child health services in Ethiopia: the role of primary health care. BMC Health Serv Res.

[6] Ethiopian central statistics agency. Ethiopian demographic health survey report; 2016. https://dhsprogram.com/pubs/pdf/FR328/FR328.

[7] Mullan Z (2016). Transforming health care in Ethiopia. Lancet Glob Health.

[8] Agnew J. Market based solutions for addressing nutritional deficiencies: The Case of Grameen Danone Foods Ltd. 2016.

[9] Yegezu R, Kitila S (2015). Assessment of factors affecting choice of delivery place among pregnant women in Jimma Zone, South West Ethiopia: cross sectional study. J Womens Health Care.

[10] Teferra AS, Alemu FM, Woldeyohannes SM (2012). Institutional delivery service utilization and associated factors among mothers who gave birth in the last 12 months in Sekela District, North West of Ethiopia: a community-based cross sectional study. BMC Pregnancy Childbirth.

[11] Bayu H, Fisseha G, Mulat A, Yitayih G, Wolday M (2015). Missed opportunities for institutional delivery and associated factors among urban resident pregnant women in South Tigray Zone, Ethiopia: a community-based follow-up study. Glob Health Action.

[12] CSA E (2013). Population projection of Ethiopia for all regions at wereda level from 2014–2017.

[13] Alemayehu S. Factors determining choice of delivery place among women’s of child bearing age in Dega Damot Woreda, West Gojjam Zone, Amhara Regional State, Ethiopia, 2014. AAU; 2014.

[14] Mokua JAB (2014). Factors influencing delivery practices among pregnant women in Kenya: a case of Wareng’District in Uasin Gishu County, Kenya. Int J Innov Sci Res.

[15] Belay A, Sendo E (2016). Factors determining choice of delivery place among women of child bearing age in Dega Damot District, North West of Ethiopia: a community based cross-sectional study. BMC Pregnancy Childbirth.

[16] Bayu H, Fisseha G, Mulat A, Yitayih G, Wolday M (2015). Missed opportunities for institutional delivery and associated factors among urban resident pregnant women in South Tigray Zone, Ethiopia: a community-based follow-up study. Glob Health Action.

[17] Ayele G, Tilahune M, Merdikyos B, Animaw W, Taye W (2015). Prevalence and associated factors of home delivery in Arbaminch Zuria district, southern Ethiopia: community based cross sectional study. Science.

[18] Kibret GD. Prevalence and Determinants of Home Birth after AnteNatal Care attendance in Gozamin District, Northwest Ethiopia. Health Science Journal 2015.

[19] Tebekaw Y, James Mashalla Y, Thupayagale-Tshweneagae G (2015). Factors influencing women’s preferences for places to give birth in Addis Ababa, Ethiopia. Obstet Gynecol Int.

[20] Tadele N, Lamaro T (2017). Utilization of institutional delivery service and associated factors in Bench Maji zone, Southwest Ethiopia: community based, cross sectional study. BMC Health Serv Res.

[21] Weldearegay H (2015). Factors affecting choice of place for childbirth among women in Ahferom Woreda, Tigray, 2013. J Pregnancy Child Health.

